# T224. THE FUNCTIONAL CONNECTIVITY DERIVED WITH BIVARIATE ANALYSIS, COHERENCE AND PHASE LOCKING VALUE IN PATIENTS WITH SCHIZOPHRENIA UNDER CLOZAPINE

**DOI:** 10.1093/schbul/sby016.500

**Published:** 2018-04-01

**Authors:** Yong Sik Kim, In Won Chung, Hee Yeong Jung, Tak Youn, Se Hyun Kim, Nam Young Lee, Seong Hoon Jeong, Kyung Tae Park, Sang Hoon Yi, Yong Min Ahn

**Affiliations:** 1 Dongguk University School of Medicine; 2 Seoul National University; 3 Eulji University Hospital; 4 Inje University

## Abstract

**Background:**

Coherence (COH) and Phase Locking Value (PLV) may have considerable potentials for investigating anomalies of functional connectivity in schizophrenia but results are still conflicting. This study is aimed to investigate relationships between plasma levels of clozapine (p-CZP) and norclozapine (p-NCZP), and total and cognitive factor scores of Positive and Negative Syndrome Scale (PANSS-T, -C), and functional connectivity by COH and PLV.

**Methods:**

Fifty-eight patients who were diagnosed as schizophrenia with DSM-5 criteria and under CZP were recruited (duration of illness, 15.5 ± 8.0 years; duration of CZP, 6.8 ± 4.6 years; mean daily dose of CZP, 233.6 ± 88.4 mg). COH and PLV were calculated with Neurophysiological Biomarker Toolbox from qEEG and were averaged from the signals of electrodes in the designated brain regions, frontal (F), temporal (T), central (C) and occipitoparietal (OP). For interhemispheric connectivity, electrodes except all midline channels were combined into Odd (O) and Even (E). The results were presented at ≥0.30 of Spearman correlation.

**Results:**

1) Correlation coefficient between p-CZP and p-NCZP was 0.84, and those of CZP dose with p-CZP and p-NCZP were 0.38 and 0.53, respectively. 2) p-CZP showed correlations with OCEC in delta and alpha, OTEC in delta, OCEOP in theta, OTEF in alpha, and OOPEF in gamma band in COH, and OOPEOP in beta band in PLV. 3) p-NCZP showed correlations with ETEOP in delta, theta, and gamma, OCEC in delta and alpha, OFOC and OCEOP in delta, OFET and OTET in alpha, OCEF in beta, OOPEC in gamma band in COH, and with ETEOP in delta, theta, and beta, OTET and OCEC in alpha, OCEF in beta band in PLV. 4) CZP dose showed correlations with ETEC in beta and gamma, ETEOP in theta, OCEF in alpha, OTET in beta, OOPEF and OOPET in gamma band in COH, and with OTET in alpha and beta, ETEOP in theta, OTEOP in alpha, ETEC in beta, OFOC in gamma band in PLV. 5) PANSS-T showed correlations with OFEOP and EFEOP in alpha, OCEOP in beta, OTOC and OTEF in gamma band in COH, and with OTEF in beta and gamma, OFET in delta, OOPEF in beta, OTOC and OCEOP in gamma band in PLV. 6) PANSS-C showed correlations with EFEOP in delta, theta, alpha, and beta, OOPEOP in delta, alpha, and beta, OFET and OTEF both in alpha and beta, OOPEF in delta, OFEOP in alpha, OFEC and OCEOP in beta, OTOC in gamma band in COH, and with EFEOP in theta, alpha, and beta, OFEOP and OOPEOP both in alpha and beta, OFET in delta and beta, OTOC, OOPEF, OOPEOP in beta, OTOC and OCEOP in PLV. 7) PANSS-T and -C showed no correlations with p-CZP, p-NCZP and CZP dose. 8) However, the clinical and drug variables showed significant simultaneous correlation with certain functional connectivity, but sometimes the direction correlation was opposite.

**Discussion:**

The relationship between functional connectivity and clozapine parameters seems to demonstrate inter- and intra-hemispheric connections in brain regions. However, there were same and/or opposite directions of correlations between COH and PLV dependent EEG band frequencies and clinical and drug variables. Taken together, investigating the functional connectivity with COH and PLV could give the information about p-CZP and p-NCZP before the laboratory reports, the degree of psychopathology in patients with schizophrenia under CZP, and the differentiations of surface symptoms whether derived from pathophysiology of schizophrenia or from clozapine effects.

